# The Persian methamphetamine use in methadone treatment in Iran: implication for prevention and treatment in an upper-middle income country

**DOI:** 10.1186/s40199-015-0134-5

**Published:** 2015-11-17

**Authors:** Zahra Alam-mehrjerdi, Mohammad Abdollahi

**Affiliations:** Program of International Research and Training, National Drug and Alcohol Research Centre, Faculty of Public Health and Community Medicine, University of New South Wales, Sydney, Australia; Department of Toxicology and Pharmacology, Faculty of Pharmacy and Pharmaceutical Sciences Research Center, Tehran University of Medical Sciences, Tehran, Iran

## Abstract

As the most populated Persian Gulf country in West Asia, methamphetamine use in methadone maintenance treatment (MMT) is a new health concern in Iran. Methamphetamine use in MMT can originate in methadone misconceptions or the stimulant effects of methamphetamine use. Several research studies have highlighted the prevalence of methamphetamine use in Iran and conducting further studies on this issue is being developed. Opiate use is treated with MMT. But, there is no effective pharmacological treatment for methamphetamine use and cognitive-behavioral interventions have still remained the best practice. As a psychostimulant drug, methamphetamine use can lead to poor treatment outcomes or even treatment failure among patients in MMT. Therefore, the implementation of methamphetamine education and prevention programs in MMT is required. Prescribing adequate methadone dose and the treatment of comorbidities as well as, doing a series of activities outside treatment is underscored. Methamphetamine use has a chronic nature and methamphetamine treatment is a long-term procedure with a high rate of relapse. Therefore, the implementation of long-term motivational interviewing, teaching necessary skills to prevent relapse and case management is highlighted. A long-term collaboration between treatment teams, patients and their families is suggested to manage methamphetamine use in MMT.

## Editorial

Methamphetamine use is a current global health concern [1]. Iran (e.g., Persia or the land of Persians) is an upper middle-income country with a population of 78 million in the west of Asia. The history of drug use in the Persian society dates back to the time of its Mesopotamia-related civilization and the Persian Empires. The use of some plants with euphoric effects was common for religious ceremonies at the time of Zoroaster and Cyrus the Great, the Persian prophet and king in Persia. Because of Afghanistan, the main global opium cultivator in the neighborhood of Iran, Afghan opiates typically opium and opium residues have been always the traditional drugs of use in Iran [2]. Nonetheless, in recent years, a manifest transition from the traditional pattern of opium use to impure methamphetamine use has been reported in the Persian society [3, 4]. Before 2005, methamphetamine use was rare in Iran [2, 3]. High purity and expensive methamphetamine was first imported from Southeast Asia. But after a few years, its production was illegally initiated with inexpensive ingredients in clandestine laboratories in Iran. This issue became a main reason for methamphetamine use in the Persian society [2, 3].

In the rapidly developing and competitive environment of Iran, when the new highly educated generation seeks for ambitious social and economic status, the stimulant effects of the Persian methamphetamine such as physical energy and wakefulness may be primarily attractive [5, 6]. Nonetheless, this transition has been primarily reported in opiate users in the society and MMT across the country [5, 6]. Literature is not well-documented on this issue. But, Iran is an exceptional country in the region that has increasingly improved its research system [7]. Therefore, conducting studies of the Persian methamphetamine use in MMT is being developed. For example; a study of 150 patients in MMT at Iranian National Center for Addiction Studies in Tehran found that 15 %, 24 %, and 12 % of the patients were methamphetamine users while in treatment in months one, three, and six, respectively [8]. A study at the methadone clinic of a central hospital in Zahedan found that methamphetamine use dramatically increased among clients from 6 % in 2009 to almost 20 % in 2011 [9]. In 2013, a survey of 7,342 men and women (82.5 % vs. 17.5 %) at methadone clinics and centers in the Northern Khorasan province found that 2.8 % of the patients were methamphetamine users in MMT [10]. A study of 120 clients at several methadone clinics in Tehran found that 64.7 % of clients smoked methamphetamine after six months of methadone treatment [11]. A recent study of oral and dental health of 95 women at women-only methadone centers in Tehran found that the mean age of the sample was 38 years old. The mean duration of drug dependence was 11.6 years. The most common drug of use was methamphetamine (71.7 %), and the most common route of methamphetamine administration was smoking [12].

Methamphetamine use in MMT has serious health implications and can originate in different reasons [1]. Some recent research studies show that methamphetamine use in MMT in Iran is associated with methadone misconceptions, relieving the side effects of methadone use such as depression and poor sexual performance, increasing physical energy, concentration, attention span, socializing with others [6], pleasure and experience seeking [13]. Methamphetamine use in MMT can be associated with other factors. A study of 150 women at a women-only methadone center in Tehran found that the median age of the sample was 34 years. Women were poly smokers of opiates with methamphetamine. Overall, 51.3 % of them had positive urine specimens for methamphetamine use in MMT. Among the women who had positive urine specimens, long duration of poly use of opiates and methamphetamine, poor family support, poor motivations to change methamphetamine use, poor participation in counseling sessions, depression, an inability to cope with everyday life pressures and poor skills to cope with methamphetamine were associated with methamphethamine use [14].

Opiate use is treated with MMT. But, methamphetamine use has no approved pharmacological treatment in the world [15]. The main treatment approach is long-term cognitive-behavioral treatment (CBT) [16] which may not be always appealing to methamphetamine users in MMT. Therefore, the implementation of methamphetamine education and prevention programs in MMT should be a health priority for treatment teams at methadone centers. The side effects of methadone use and/or methadone misconceptions should be gradually explained and settled down by treatment teams at methadone centers. The collaboration of medical doctors with psychologists at the centers is required to eliminate these problems. Methamphetamine use in MMT may lead to treatment failure and the sudden interruption of methadone program with no definite positive outcome. Therefore, the simultaneous provision of psychological interventions such as CBT and prescribing adequate methadone dose are primarily underscored. Adequate physical exercise, having an appropriate nutrition, a practical program for daily activities and strong family relationships and support should be simultaneously consulted with clients and their families. Psychiatric and physical comorbidities should be specifically addressed. Comorbidities such as decreased sexual desire and depression may increasingly facilitate methamphetamine use in MMT. Most of the clients may not report craving for methamphetamine use in MMT because using regular methadone dose could prevent drug craving in general. Such clients may use methamphetamine because of their former addictive life styles, peer pressure and living in environments with an easy access to inexpensive methamphetamine. Methamphetamine use has a chronic nature and methamphetamine treatment is a long-term procedure with a high rate of relapse [2–4]. Therefore, the implementation of long-term motivational interviewing and teaching necessary skills to prevent relapse and case management is suggested.
